# Overexpression of IL-17RC associated with ocular sarcoidosis

**DOI:** 10.1186/1479-5876-12-152

**Published:** 2014-05-31

**Authors:** Wenting Wu, Ming Jin, Yujuan Wang, Baoying Liu, Defen Shen, Ping Chen, Susan Hannes, Zhiyu Li, Sima Hirani, Shayma Jawad, H Nida Sen, Chi-Chao Chan, Robert B Nussenblatt, Lai Wei

**Affiliations:** 1 Laboratory of Immunology, National Eye Institute, National Institutes of Health, Bethesda, MD 20892, USA; 2 Beijing University of Chinese Medicine, Beijing, China; 3 Department of Ophthalmology, China-Japan Friendship Hospital, Beijing, China; 4 State Key Laboratory of Ophthalmology, Zhongshan Ophthalmic Center, Sun Yat-sen University, Guangzhou, China

**Keywords:** Ocular sarcoidosis, IL-17RC, CD8

## Abstract

**Background:**

Sarcoidosis is a chronic inflammatory disease with a systemic granulomatous disorder affecting multiple organs including the eye. Both CD4^+^ T cell and macrophage have been linked to the pathogenesis of the disease.

**Methods:**

The expression of IL-17RC was measured using FACS,immunohistochemistry and real-time PCR. Serum level of IL-17 was detected using ELISA.

**Results:**

An elevated expression of IL-17RC on CD8^+^ T cells in peripheral blood was found in patients with ocular sarcoidosis as compared to healthy controls. Interestingly, we found a significant increase in the serum level of IL-17 in patients with ocular sarcoidosis as compared to healthy controls, which may be responsible for the induction of IL-17RC on CD8^+^ cells. In addition, IL-17RC appeared only in the retinal tissue of the patient with clinically active sarcoidosis.

**Conclusions:**

Our results suggested a potential involvement of IL-17RC^+^CD8^+^ T cells in pathogenesis of ocular sarcoidosis.

## Background

Sarcoidosis is a systemic granulomatous disorder affecting multiple organs. Chronic inflammation occurs predominantly in the lung. In addition, lymph node, gastrointestinal tract, skin, nervous system, liver, spleen, heart, kidney, and muscle can also be affected. Ocular involvement is found in 25-50% patients with sarcoidosis and can be the first clinical manifestation of the disease according to several American and European studies [1,2]. However, ocular involvement may be found in up to 89% of Japanese patients with systemic sarcoidosis, suggesting a potential racial difference of its clinical presentation. Ocular sarcoidosis can occur in the absence of other systemic manifestations. Although the diagnosis of sarcoidosis can be confirmed by biopsy of the skin, conjunctiva, peripheral lymph nodes, or lung, the etiology of sarcoid granulomas is unknown [1].

Recent studies suggest sarcoidosis susceptibility is dependent on both genetic and environmental risk factors [3]. All races can suffer from sarcoidosis. Aggregation among certain racial/ethnic groups [4], as well as increased concordance among monozygotic twins [5] both strongly support the probability of a genetic susceptibility to sarcoidosis. Recent case–control genetic studies and genome-wide association studies (GWAS) identified multiple single nucleotide polymorphisms (SNPs) that may be associated with sarcoidosis. These include polymorphisms within coding regions of *HLA-DRB1* and *HLA-DQB1*, *CARD15* (caspase recruitment domain family member 15)/*NOD2* (nucleotide-binding oligomerization domain containing 2), *BTNL2* (butyrophilin-like protein 2), *ANXA11* (annexin A11), *RAB23* (RAB23, member RAS oncogene family), and *CFH* (complement factor H) [6-14]. Moreover, exposure to musty odors and insecticides [15], as well as infectious agents including *Propionibacterium acnes* and *Mycobacterium tuberculosis*[16-18] found within sarcoid granulomas have been proposed as important environmental and occupational risk factors for sarcoidosis. Therefore, it is postulated that sarcoidosis is a multifactorial disease triggered by environmental and infectious agents in genetically susceptible individuals. However, it is unclear whether the different clinical manifestations presented in sarcoidosis patients are due to the unique etiology. For example, no clear genetic and environmental risk has been identified for ocular sarcoidosis yet. Importantly, current therapeutic strategies for sarcoidosis (such as corticosteroids, methotrexate/azathioprine/mycophenolate mofetil, and anti-TNFα treatment) were not designed according to any of the recently identified genetic and environmental risk factors.

Although the precise cause of inflammation in sarcoidosis remains an enigma, it is well-established that CD4^+^ T helper cells and macrophages play critical roles during granuloma formation [19]. In addition to CD4^+^ T cells, CD8^+^ T cells have also been found in granulomas and BAF [20,21], while its role in the pathogenesis of sarcoidosis is unclear. Locally and systemically activated macrophages release cytokines and chemokines such as IL-1β, IL-6, IL-23, IL-12, IL-18, and CCL20, leading to the recruitment of IL-17 producing T helper 17 (Th17) cells to granulomas [22-25]. IL-17 is physiologically critical during host defense against bacterial, mycobacterial, and fungal infections [26]. However, IL-17 producing Th17 cells have been the leading cause of pathological inflammation in many diseases such as multiple sclerosis and rheumatoid arthritis [27]. Recent studies have found a predominant increase of IL-17A^+^CD4^+^ memory T cells in the peripheral blood and bronchoalveolar lavages of pulmonary sarcoidosis patients [22,24], strongly suggesting (Th17) cells may play an important role in the pathogenesis of sarcoidosis.

Our previous study has suggested an elevated IL-17RC expression on several types of cells in the peripheral blood and retinal tissues from patients with Age-related Macular Degeneration [28]. Therefore, in this study, we explored whether the expression of IL-17RC was changed in ocular sarcoidosis patients.

## Methods

### Patients

All protocols were approved by institutional review boards, and written informed consents were provided by the patients to the National Institutes of Health. The patient information including disease activity and medications was listed in Table 1. All patients present ocular manifestations. 10 of these 13 patients were biopsy proven; 3 of them were with active disease when blood samples were drawn; 4 of these 13 patients had sarcoid manifestations involving in organs other than the eye. The majority of these patients were on medications (listed in Table 1).

**Table 1:** Patient information

	**Sarcoidosis (Total 13)**	**Control (Total 18)**
***General information***		
Gender (M/F)	5/8	13/5
Ave. Age	44	43
Race (AA/C/H*)	11/2/1	18/0
Ocular sarcoid	13 (100%)	-
Neuro/Pulmonary/Joint Sarcoid	1(8%)/2 (15%)/1(8%)	-
Active disease	3 (23%)	-
Biopsy proven	10 (77%)	-
Medication		
Corticosteroid**	10 (77%)	-
Immunosuppressive agent***	7 (54%)	-
Opioid	4 (31%)	-
Hypertension drug	3 (23%)	-
Heart disease drug	3 (23%)	-
Diabetic Medication	4 (31%)	-
Calcium	4 (31%)	-

*AA African American; *C* Caucasian; *H* Haitian.

**Include Prednisone, Prednisolone Acetate, Difluprednate, Desonide, Retisert.

***Include mycophenolate Mofetil, Cyclosporine, Methotrexate.

### Detection of cell surface molecules by flow cytometry

Whole blood, collected in sodium-heparin vacutainers (BD, CA), from patients or healthy volunteers was first subjected to red blood cell lysis using ACK lysis buffer (Quality Biological Inc, MD). Cells were then incubated in sterile PBS supplemented with 2% FCS and fluorochrome-conjugated antibodies against CD3, CD4, CD8, CD14, CD19, CD56 (all from BD, CA) and IL-17RC (FAB22691A) and mouse-IgG_2B_ isotype control (both from R&D, MN), for 30 minutes at room temperature in the dark. Cells were washed three times and analysed on a MACSQuant flow cytometer (Miltenyi Biotec, Germany). All flow cytometry data were analysed using FlowJo 7.6 (Treestar, OR).

### ELISA

Sera from sarcoidosis patients and healthy controls were centrifuged at 1400 rpm for 15 min after 30 min of clotting and were stored at -80°C, followed by detection of IL-17A using the Human IL-17 Quantikine ELISA Kit (R&D Systems, Minneapolis, MN) according to the manufacture’s protocol.

### Cell culture

CD8^+^ T cells were first isolated from peripheral blood of healthy controls or Sarcoidosis patients using EasySep Human CD8 Positive Selection Kit (StemCell Technologies, Canada). The cells were then cultured in RPMI-1640 medium supplemented with 10% fetal bovine serum (FBS), 2 mM L-glutamine, 1X penicillin-streptomycin antibiotics (Invitrogen, CA), and stimulated by plate-bound anti-CD3 (5 ng/ml) and anti-CD28 (2 ng/ml) antibodies (both from eBioscience, CA), with or without IL-17A (100 ng/ml) for 3 days.

### Immunohistochemistry

Immunohistochemistry was performed as previously described [28]. Briefly, the citrate retrieval and avidin-biotin-complex immunoperoxidase technique was utilized on the unstained, de-paraffinized slides of two eyes from two patients with clinically active or quiet sarcoidosis. The primary antibody was rabbit anti-human IL-17RC polyclonal antibody (sc-99937) (Santa Cruz Biotechnology, CA) or control rabbit IgG. The secondary antibody was biotin-conjugated goat anti-rabbit IgG (Vector Laboratories, Burlingame, CA). The substrate was avidin-biotin-peroxidase complex (Vector Laboratories, Burlingame, CA), and the chromogen was diaminobenzidine and nickel sulfate. The positive reaction will result in the production of a brown-blackish color.

### Microdissection and RT-PCR

Microdissection of retinal tissues and RT-PCR assays for *IL17RC* expression were conducted as previously described [27]. Briefly, retinal tissues with either granulomatous inflammation (inflammatory) or non-inflammatory (normal) areas were microdissected. Total RNA was isolated using the Paradise RNA isolation kit (Applied Biosystems, CA). SYBR Green (Qiagen) primers were used for human *IL17RC* detection. All data were normalized to the *beta-actin* mRNA level. Expression fold-change was calculated by 2^-ΔΔCT^.

### Statistical analysis

Statistical analysis was performed using GraphPad Prism 5.0 software (GraphPad, CA). The Mann–Whitney test was used to compare differences between two groups and the significance level was set at P < 0.05.

## Results

### Elevated expression of IL-17RC on CD8^+^ T cells in peripheral blood of patients with ocular sarcoidosis

**Figure 1:**
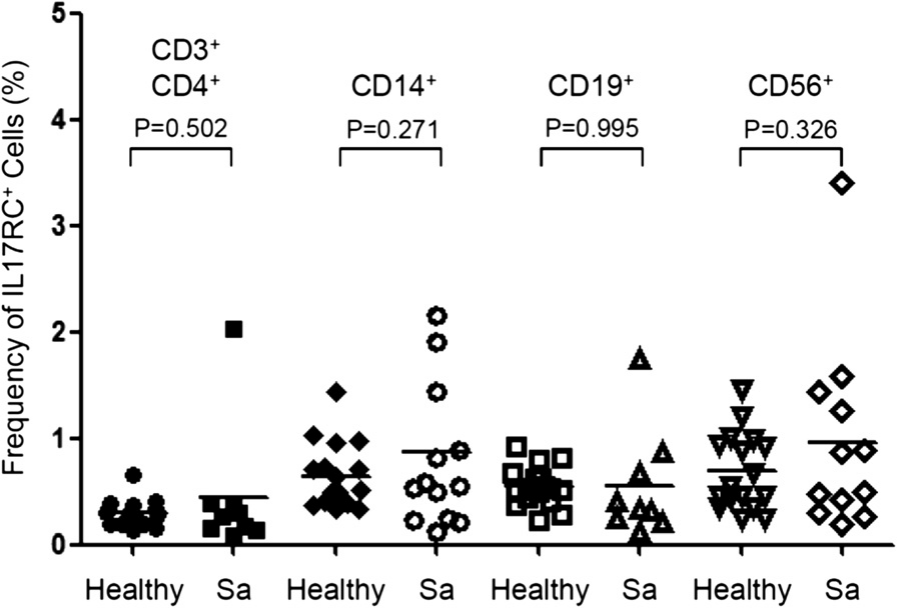
**Frequency of IL-17RC**^**+ **^**cells among peripheral blood cells.** The frequency of IL-17RC^+^CD3^+^CD4^+^ T cells, IL-17RC^+^CD14^+^ monocytes, IL-17RC^+^CD19^+^ B cells, and IL-17RC^+^CD56^+^ NK cells among each cell types in the peripheral blood from 16 healthy controls (Healthy) and 13 patients with ocular sarcoidosis (Sa) was shown.

**Figure 2:**
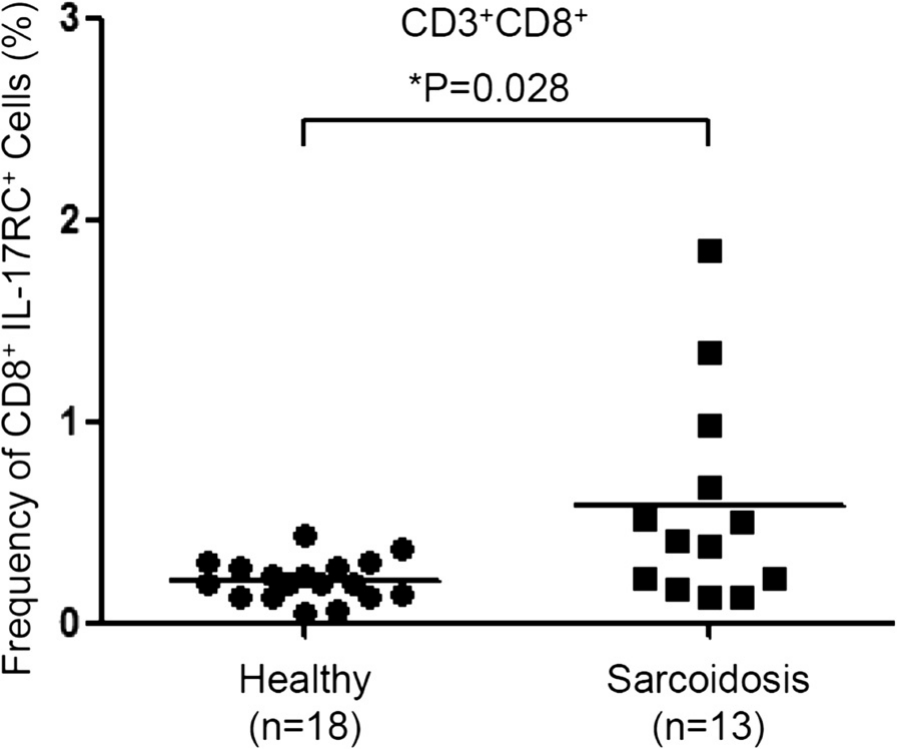
**Frequency of IL-17RC**^**+**^**CD8**^**+ **^**T cells.** The frequency of IL-17RC^+^CD3^+^CD8^+^ T cells among peripheral CD8^+^ T cells from 18 healthy controls and 13 patients with ocular sarcoidosis.

Our previous study identified a hypomethylated *IL17RC* promoter and elevated expression of IL17RC on CD14^+^ monocytes in the peripheral blood of patients with age-related macular degeneration [28]. Sarcoidosis has been suggested as a Th17 disease [19] and IL17RC is critical in mediating IL-17 induced tissue damage [29]. Therefore, we first tested whether the expression of IL-17RC was increased on PBMCs from patients with ocular sarcoidosis. FACS analysis of cell surface IL-17RC expression and markers identifying several major types of cells among PBMCs were performed. Intriguingly, the frequency of IL-17RC^+^ cells among CD3^+^CD4^+^ helper T cells, CD14^+^ monocytes, CD19^+^ B cells, and CD56^+^ NK cells was similar between healthy controls and ocular sarcoidosis patients (Figure 1). Importantly, we found that IL-17RC expression was significantly elevated only in CD8^+^ T cells in sarcoidosis patients as compared to healthy controls (Figure 2), suggesting a potential role the IL-17RC^+^CD8^+^ T cells may play in the pathogenesis of ocular sarcoidosis.

### Induction of IL-17RC expression by IL-17A in activated CD8^+^ T cells

**Figure 3:**
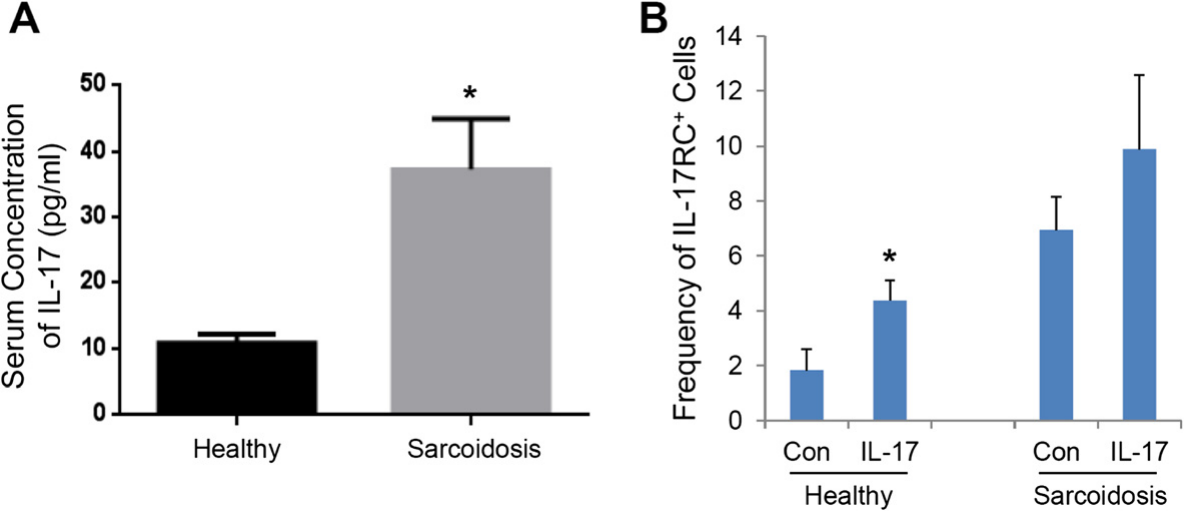
**Induction of IL-17RC on CD8**^**+ **^**T cells. (A)** Serum levels of IL-17 in 18 healthy controls and 13 patients with ocular sarcoidosis. **(B)** The frequency of IL-17RC^+^ in cultured CD8^+^ T cells treated with (IL-17) or without (Con) IL-17 from 4 healthy controls and 4 sarcoidosis patients. *P < 0.05.

Very few cells were found to express cell-surface IL-17RC in the peripheral blood of healthy volunteers. It is unclear how IL-17RC was induced in CD8^+^ T cells in sarcoidosis patients. Our previous study suggested that IL-17, the ligand of IL-17RC, can induce IL-17RC expression in human monocyte and retinal pigment epithelium (RPE) [28]. Importantly, we found significantly elevated levels of IL-17 in the serum of patients with ocular sarcoidosis (Figure 3A). Therefore, we next examined whether IL-17 can induce the expression of IL-17RC on CD8^+^ T cells. CD8^+^ T cells were isolated from peripheral blood of healthy controls or Sarcoidosis patients and stimulated by plate-bound anti-CD3 and anti-CD28 antibodies with or without IL-17 for 3 days. As shown in Figure 3B, IL-17 significantly increased the expression of IL-17RC in the culture of peripheral CD8^+^ T cells from healthy controls. Consistent with the above results that an elevated expression of IL-17RC was found on CD8^+^ T cells in peripheral blood from patients with ocular sarcoidosis (Figure 2), more IL-17RC^+^ cells were found in the cultures of CD8^+^ T cells, stimulated by plate-bound anti-CD3 and anti-CD28 antibodies for 3 days, from sarcoidosis patients than in the culture of CD8^+^ T cells from healthy controls (Figure 3B). However, we only found a minimal induction of IL-17RC in response to IL-17 treatment on CD8^+^ T cells from sarcoidosis patients (Figure 3B).

### Elevated expression of IL-17RC in the eyes from patients with clinically active ocular sarcoidosis

**Figure 4:**
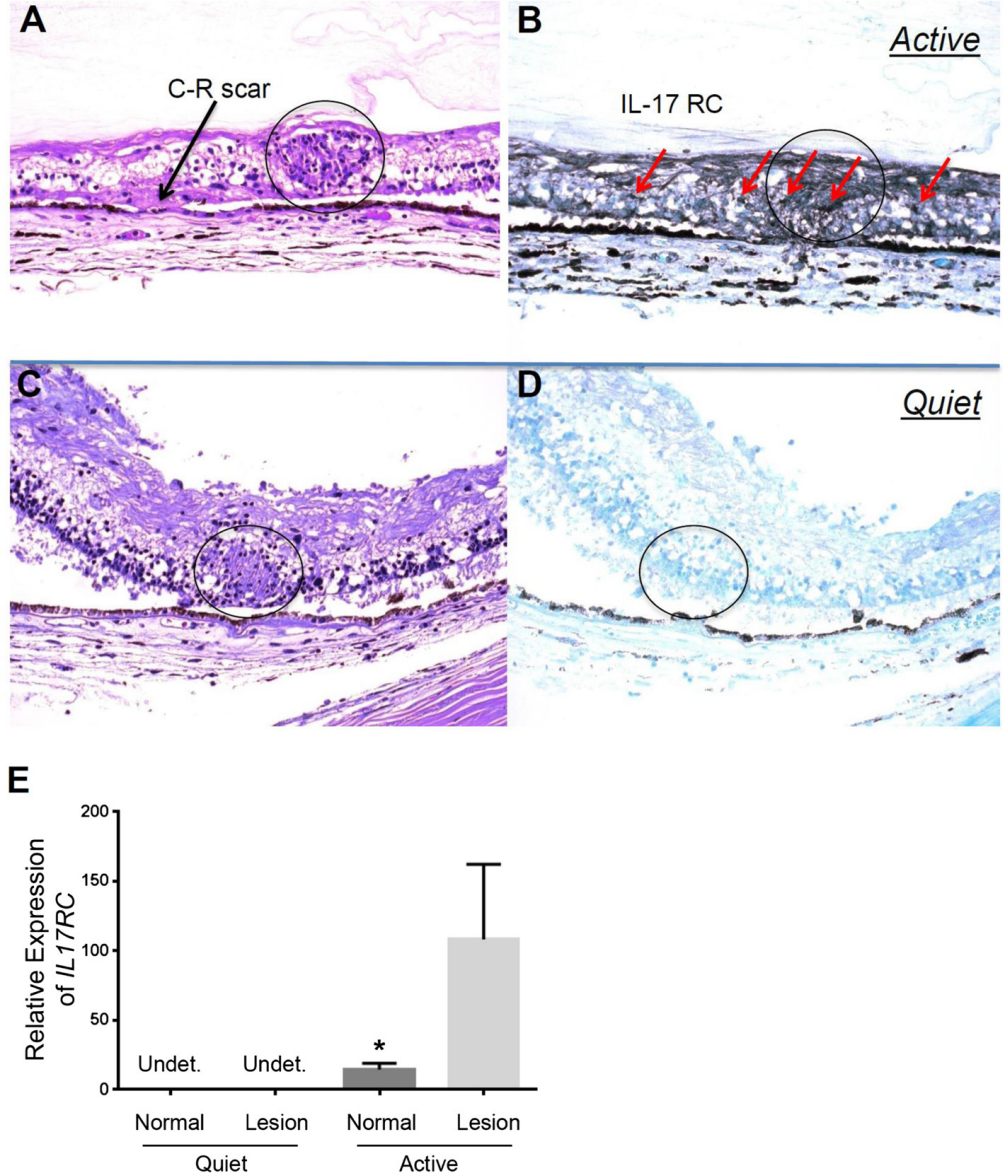
**Expression of IL-17RC in the retinal tissue of patients with active or quiet ocular sarcoidosis.** HE staining **(A)** and immunohistochemistry staining of IL-17RC protein in the retinal tissue of an active sarcoidosis patient **(B)** as well as HE staining **(C)** and Immunohistochemistry staining of IL-17RC protein in the retinal tissue of a quiet sarcoidosis patient **(D)** were shown. The expression of IL17RC in the microdissected normal or lesion retinal tissues from patients with quiet or active ocular sarcoidosis **(E)** were shown. Circle, granuloma; Red arrow, IL-17RC^+^ cells.

Previous studies have suggested that IL-17RC^+^ cells may be able to migrate to the eye [28], we therefore tested whether IL-17RC^+^ cells could be found in the retinal tissues of patients with either active or quiet ocular sarcoidosis. As shown in Figure 4A-D, immunohistochemistry assays demonstrated that IL-17RC expression in the retinal tissues of patients with clinically active ocular sarcoidosis (Figure 4A and B) was higher than its expression in the eye from patients with quiet disease clinically but small focal inflammation histopathologically (Figure 4C and D). In addition, in the eye with clinically active sarcoidosis, *IL17RC* RNA expression averaged at 108.1 fold higher in the retinal inflammatory lesion but only 14.2 fold higher in the normal retina compared to the normalization with *beta-actin* mRNA level. However, *IL17RC* expression was below the detectable level in both the inflammatory and normal retina of the eye with clinically quiet disease (Figure 4E). Taken together, these data suggest that the expression of IL-17RC may correlate with the clinical disease severity.

## Discussion

Both Th1 and Th17 cells have been found to be crucial in leading to the formation of granulomas, the clinical hallmark of sarcoidosis [22]. An elevated level of IL-17 has been found in granulomas in the lung, BAF, as well as circulating CD4^+^ T cells in patients with pulmonary sarcoidosis [24,30]. In our study, we also found a significant increase in the serum level of IL-17 in patients with ocular sarcoidosis as compared to healthy controls. Therefore, IL-17 mediated chronic inflammation may be a leading cause of tissue pathology in sarcoidosis.

Our previous study suggests that IL-17 is able to upregulate the expression of its own receptor, IL-17RC, on multiple cell types including CD14^+^ monocytes and retinal pigment epithelium to amplify its biological effects [28]. In our current study, we also found that IL-17 can induce the expression of IL-17RC on peripheral CD8^+^ T cells. Therefore, the elevated expression of IL-17RC in the peripheral blood of patients with ocular sarcoidosis may be due to the effect of increased serum level of IL-17 in these patients. However, as shown in Figure 1, the frequency of IL-17RC^+^ monocytes and NK cells was also slightly increased, without a statistical significance probably due to the small sample size. Therefore, elevation in the expression of IL-17RC may not be CD8^+^ T cell specific. It could be a common response of multiple cell types in the peripheral blood due to the elevated level of IL-17. Importantly, we found sustained expression of IL-17RC protein and transcripts in the retinal tissue with granulomatous inflammation of clinically active but not quiet sarcoidosis patients. These results suggested that IL-17RC^+^ cells may be able to migrate into the eye and facilitate the formation of granulomas.

CD8^+^ T cells, in addition to CD4^+^ T cells, have also been found in granulomas [21]. We have also reported that 10% of the lymphocytic infiltration was CD8^+^ T cells but these cells were rarely found within granulomas in an eye with clinically active sarcoidosis [31]. Candia et al. [32] as well as Zhou et al. [33] have also suggested a crucial role CD8^+^ T cells play in the pathogenesis of sarcoidosis. In this study, we found that the frequency of IL-17RC^+^CD8^+^ cells was increased in ocular sarcoidosis patients, which strongly suggests involvement of inflammatory CD8^+^ T cells in sarcoidosis. Many infectious pathogens have been found in granulomas in sarcoidosis patients. It would be interesting to investigate whether evidence of viral infection can be identified in or around granulomas given the potential involvement of CD8^+^ T cells.

A low frequency of IL-17RC^+^ cells in the whole blood was found on CD8^+^ T cells, not only in healthy controls but also in patients with ocular sarcoidosis. However, a significantly more IL-17RC^+^ cells can be found in the cultures of CD8^+^ T cells stimulated by anti-CD3/anti-CD28 antibodies as compared to the unstimulated whole blood test, suggesting that during active inflammation, IL-17RC^+^CD8^+^ T cells might be activated by local antigen in the eye, which might contribute to the formation of granulomas in sarcoidosis patients.

## Conclusions

Our results demonstrated an elevated expression of IL-17RC on CD8^+^ T cells in patients with sarcoidosis, supporting a potential role of these T cells played in the pathogenesis of ocular sarcoidosis.

## Competing interests

The authors declare that they have no competing interests.

## Authors' contributions

LW and RBN conceived the study and drafted the manuscript. LW, MJ, HNS, CCC, and RBN participated in the study design. WW, YW, PC, BL, ZL, SH, and SJ carried out the experiments. All authors read and approved the final manuscript.
